# Supplementary material for: Psychometric Performance of the Stony Brook Scar Evaluation Scale and SCAR-Q Questionnaire in Dutch Children after Pediatric Surgery
Source: Int J Environ Res Public Health. 2023 Dec 30;21(1):57. doi: 10.3390/ijerph21010057 (PMC10815752; doi:10.3390/ijerph21010057)
Supplement: Supplementary file 1 [file ijerph-21-00057-s001.zip › ijerph-2723208-supplementary.pdf]

Supplementary material of the manuscript:

Psychometric Performance of the Stony Brook Scar Evaluation Scale and SCAR-Q Questionnaire in Dutch Children after Pediatric Surgery

Chantal A. Ten Kate [1] , Hilde J. H. Koese [1], M. Jenda Hop [2], André B. Rietman [1,3] , René M. H. Wijnen [1], Marijn J. Vermeulen [4] and Claudia M. G. Keyzer-Dekker [1,*]

[1] Department of Pediatric Surgery and Intensive Care Children, Erasmus MC Sophia Children's Hospital, Wytemaweg 80, 3015 CD Rotterdam, The Netherlands; c.tenkate@erasmusmc.nl (C.A.T.K.); hildekoese@gmail.com (H.J.H.K.); a.rietman@erasmusmc.nl (A.B.R.); r.wijnen@erasmusmc.nl (R.M.H.W.)
[2] Department of Plastic and Reconstructive Surgery, Erasmus University Medical Center, 3015 CD Rotterdam, The Netherlands; m.hop@erasmusmc.nl
[3] Department of Child and Adolescent Psychiatry/Psychology, Erasmus MC Sophia Children's Hospital, 3015 CD Rotterdam, The Netherlands
[4] Department of Pediatrics, Division of Neonatology, Erasmus MC Sophia Children's Hospital, 3015 CD Rotterdam, The Netherlands; m.j.vermeulen@erasmusmc.nl
* Correspondence: c.keyzer-dekker@erasmusmc.nl; Tel.: +31-(0)-10-70-36240

Contents:

S1. Supplementary methods

S2. POSAS results

S3. Item evaluation, reliability and validity of SBSES and SCAR-Q

S4. Influence of passed time since surgery

S1. Supplementary methods

Stony Brook Scar Evaluation Scale (SBSES)[9]

The SB is a pragmatic 5-point observer-report suitable for the photographic evaluation of linear scars It assesses the following aspects: width (>2 mm or <2 mm), height (elevated/depressed or flat), color (darker or same color/lighter), hatch/suture marks (present or absent), and overall appearance (poor or good). Each item can be assigned a score 0 or 1, and missing items are not allowed. The total score is calculated as the sum of the scores for the five items, resulting in a score that can range from 0 (worst) and 5 (best).

SCAR-Q questionnaire[8]

The SCAR-Q is a self-report questionnaire for children aged 8-17 years old. It comprises three domains: an appearance scale (12 items), a symptom scale (12 items), and a psychosocial impact scale (5 items). All items can be scored on a 4-point Likert scale, ranging from 'doesn't bother at all' to 'bothers very much'. If <50% of the items are missing, the mean of the completed items can be used to estimate these missing item scores. If ≥50% of the items are missing, total scores cannot be calculated. Total scores are determined for each domain by transforming the sum score, using an equivalent Rasch analysis, resulting in scores that range from 0 (worst) to 100 (best).

Patient and Observer Scar Assessment Scale[7]

The POSAS is an observer-report tool designed for the onsite evaluation of both linear and burn scars by a physician. It comprises seven items: vascularity, pigmentation, thickness, relief, pliability, surface area, and overall opinion. Each item can be rated on a scale from 1 (normal skin) to 10 (worst scar imaginable). For each item, a description can be added to provide further details. For vascularity, categories such as pale, pink, red, purple or mix can be specified. Pigmentation can be described as hyper, hypo or mix. Thickness may be specified as thicker or thinner. Relief can be noted as more, less or mix. Pliability can be assessed as supple, stiff or mix. Surface area can be characterized as expansion, contraction, or mix. All items are preferably compared to normal skin at a comparable anatomic location. Last, an overall opinion of the scar is given, with a score ranging from 1 (normal skin) to 10 (worst scar imaginable). The total score is calculated as the sum of the six items, resulting in a range from 6 (best) to 60 (worst).

Standardized protocol of scar photography

After visiting the outpatient clinic, the patient was referred to the hospital's medical photographer. An application form, filled out by the research team at the outpatient clinic, provided information about the location of the surgical scar(s) on the body. In cases of multiple surgical scars, all scars were photographed. For each scar, two images were captured. The first image was taken at a 90-degree angle and included a ruler to indicate the length of the scar, while the second image was taken at a 45-degree angle to capture the scar's depth. Both images were screen-filing. All images were taken using a Nikon D750 digital camera equipped with a 105mm lens (Nikon Corporation, Minato, Tokyo, Japan).

The camera settings included an exposure time 1/125sec, a diaphragm setting of. 22-25, white balance set D1, and flash mode between 8.2 and 9.2.

Translation of instruments

The POSAS and SCAR-Q were provided in the Dutch language by the developers. The SB underwent an English-Dutch forward-backward translation process following the principles outlined in 'Translation and Cultural Adaptation of Patient Reported Outcomes Measures - Principles of Good Practice'[13]. This process involved two independent forward translations by native Dutch-speaking translators and one backward translation by a native Swedish-speaking translator. All translations were reviewed by the research team until consensus was reached, with documentation of all reconciliation decisions.

S2. POSAS results

Parameter	Median (range)	n (%)
Vascularity	3 (1-5)	
Pale		68 (68.0)
Pink		9 (9.0)
Red		15 (15.0)
Purple		0 (0.0)
Mix		0 (0.0)
N/A		8 (8.0)
Pigmentation	3 (1-6)	
Hypo		68 (68.0)
Hyper		20 (20.0)
Mix		0 (0.0)
N/A		12 (12.0)
Thickness	3 (1-8)	
Thicker		51 (51.0)
Thinner		36 (36.0)
N/A		13 (13.0)
Relief	2.5 (1-7)	
More		60 (60.0)
Less		13 (13.0)
Mix		2 (2.0)
N/A		25 (25.0)
Pliability	3 (1-7)	
Supple		8 (8.0)
Stiff		79 (79.0)
N/A		13 (13.0)
Surface area	2 (1-7)	
Expansion		50 (50.0)
Contraction		4 (4.0)
Mix		2 (2.0)
N/A		44 (44.0)
Overall opinion	3 (1-7)	
Total score	15 (8-33)	

Supplementary Table S1. Results of the Patient and Observers Scar Assessment Scale (POSAS, n=100). Item scores range from 0 to 10, total score range from 6 to 60. N/A = not applicable, when the parameter is scored as 1 (normal skin).

S3. Item evaluation, reliability and validity of the SBSES and SCAR-Q

Individual item	Missing values, n (%)	Mean ± SD
Observer 1		
Width	0 (0)	0.68 ± 0.47
Height	0 (0)	0.86 ± 0.35
Color	0 (0)	0.63 ± 0.49
Suture marks	0 (0)	0.67 ± 0.47
Overall opinion	0 (0)	0.71 ± 0.46
Observer 2		
Width	0 (0)	0.35 ± 0.48
Height	0 (0)	0.56 ± 0.50
Color	0 (0)	0.61 ± 0.49
Suture marks	0 (0)	0.64 ± 0.48
Overall opinion	0 (0)	0.57 ± 0.50
Observer 3		
Width	0 (0)	0.60 ± 0.49
Height	0 (0	0.72 ± 0.45
Color	0 (0)	0.69 ± 0.47
Suture marks	0 (0)	0.79 ± 0.41
Overall opinion	0 (0)	0.60 ± 0.49

Supplementary Table S2. Item evaluation of the Stony Brook Scar Evaluation Scale (SBSES) by three surgeons, who each assessed 100 scars. Raw scores are presented. Items could be scored 0 (worst) to 1 (best).

Individual item		Missing values, n (%)	Mean ± SD
Appearance scale			
**Q1**	Far away	0 (0)	3.43 ± 0.81
**Q2**	Width	0 (0)	3.48 ± 0.86
**Q3**	Different angles	0 (0)	3.30 ± 0.87
**Q4**	Thickness	0 (0)	3.57 ± 0.89
**Q5**	Length	0 (0)	3.13 ± 0.98
**Q6**	Bumpy	0 (0)	3.54 ± 0.81
**Q7**	Color	0 (0)	3.61 ± 0.75
**Q8**	Difference from skin	0 (0)	3.52 ± 0.78
**Q9**	Contour	0 (0)	3.57 ± 0.72
**Q10**	Overall size	1 (1)	3.24 ± 0.96
**Q11**	Up close	0 (0)	3.13 ± 0.93
**Q12**	Noticeable	0 (0)	3.04 ± 1.03
Symptom scale			
**Q1**	Dry	0 (0)	3.80 ± 0.50
**Q2**	Hard to move	0 (0)	3.78 ± 0.51
**Q3**	Painful	0 (0)	3.50 ± 0.78
**Q4**	Puffy	0 (0)	3.83 ± 0.57
**Q5**	Tingly	0 (0)	3.76 ± 0.48
**Q6**	Numb	0 (0)	3.43 ± 0.81
**Q7**	Pulling	0 (0)	3.37 ± 1.04
**Q8**	Do activities	0 (0)	3.67 ± 0.73
**Q9**	Itchy	0 (0)	3.65 ± 0.64
**Q10**	Sensitive	0 (0)	3.67 ± 0.70
**Q11**	Hard or firm	0 (0)	3.41 ± 0.86
**Q12**	Tight	0 (0)	3.54 ± 0.75
Psychosocial impact			
**Q1**	People see	0 (0)	3.30 ± 0.87
**Q2**	Embarrassed	0 (0)	3.63 ± 0.80
**Q3**	Upset	0 (0)	3.54 ± 0.81
**Q4**	Dislike seeing	0 (0)	3.54 ± 0.91
**Q5**	Unhappy	0 (0)	3.63 ± 0.80

Supplementary Table S3. Item evaluation of the SCAR-Q (n=46). Raw, untransformed scores are presented. Items could be scored 1 (bothers very much) to 4 (does not bother at all). One child omitted one item.

External reliability (interobserver)		Level of agreement, ICC (95% CI)
	Width	
	Observer 1 vs. observer 2	0.29 (0.06-0.49)
	Observer 1 vs. observer 3	0.52 (0.37-0.66)
	Observer 2 vs. observer 3	0.38 (0.17-0.55)
	Height	
	Observer 1 vs. observer 2	0.21 (0.02-0.39)
	Observer 1 vs. observer 3	0.36 (0.18-0.52)
	Observer 2 vs. observer 3	0.45 (0.28-0.60)
	Color	
	Observer 1 vs. observer 2	0.49 (0.33-0.63)
	Observer 1 vs. observer 3	0.69 (0.57-0.78)
	Observer 2 vs. observer 3	0.70 (0.58-0.79)
	Suture marks	
	Observer 1 vs. observer 2	0.67 (0.55-0.77)
	Observer 1 vs. observer 3	0.45 (0.28-0.60)
	Observer 2 vs. observer 3	0.50 (0.33-0.64)
	Overall opinion	
	Observer 1 vs. observer 2	0.45 (0.28-0.59)
	Observer 1 vs. observer 3	0.37 (0.19-0.53)
	Observer 2 vs. observer 3	0.41 (0.23-0.56)

External reliability (intraobserver)		Level of agreement, ICC (95% CI)
	Width	0.70 (0.58-0.79)
	Height	0.57 (0.42-0.69)
	Color	0.75 (0.65-0.83)
	Suture marks	0.69 (0.57-0.78)
	Overall opinion	0.56 (0.41-0.68)

		Scar length		Scar width	
		r_{pb}	*p-value*	r_{pb}	*p-value*
Criterion validity	Observer 1				
	Width	-0.18	0.08	-0.40	<0.001
	Height	-0.03	0.77	0.02	0.85
	Color	0.09	0.35	0.10	0.32
	Suture marks	0.06	0.57	-0.04	0.71
	Overall score	0.02	0.88	-0.24	0.02
	Observer 2				
	Width	-0.11	0.29	-0.21	0.04
	Height	-0.15	0.13	-0.15	0.15
	Color	0.07	0.50	-0.02	0.85
	Suture marks	0.08	0.45	-0.06	0.58
	Overall score	-0.19	0.06	-0.33	<0.001
	Observer 3				
	Width	-0.20	0.04	-0.37	<0.001
	Height	-0.31	0.002	-0.15	0.14
	Color	0.10	0.31	0.11	0.26
	Suture marks	-0.16	0.11	-0.08	0.45
	Overall score	-0.20	0.04	-0.23	0.02

Convergent validity		Level of agreement, r_s (95% CI)
	Color vs. vascularity	
	Observer 1 vs. POSAS	-0.36 (-1.10- -0.37)
	Observer 2 vs. POSAS	-0.23 (-0.87- -0.11)
	Observer 3 vs. POSAS	-0.34 (-1.10- -0.33)
	Height vs. thickness	
	Observer 1 vs. POSAS	-0.11 (-1.68- -0.09)
	Observer 2 vs. POSAS	-0.17 (-1.06- 0.06)

	Observer 3 vs. POSAS	-0.33 (-0.83- 0.00)
	Overall score vs. overall impression	
	Observer 1 vs. POSAS	-0.28 (-0.46- -0.09)
	Observer 2 vs. POSAS	-0.23 (-0.40- -0.03)
	Observer 3 vs. POSAS	-0.51 (-0.64- -0.35)
		**Level of agreement, r_s (95% CI)**
**Convergent validity**	SCARQ – appearance scale	
	Vascularity	-0.23 (-0.49- 0.07)
	Pigmentation	-0.10 (-0.38- 0.19)
	Thickness	-0.09 (-0.37- 0.20)
	Relief	-0.18 (-0.45- 0.12)
	Pliability	-0.10 (-0.38- 0.20)
	Surface	-0.09 (-0.37- 0.20)
	Overall impression	-0.10 (-0.38- 0.19)
	SCARQ - symptom scale	
	Vascularity	-0.36 (-0.59- -0.07)
	Pigmentation	-0.05 (-0.34- 0.24)
	Thickness	-0.18 (-0.44- 0.12)
	Relief	0.08 (-0.22- 0.36)
	Pliability	-0.19 (-0.46- 0.11)
	Surface	0.10 (-0.20- 0.38)
	Overall impression	-0.05 (-0.34- 0.24)
	SCARQ psychosocial scale	
	Vascularity	-0.24 (-0.50- 0.05)
	Pigmentation	-0.07 (-0.35- 0.23)
	Thickness	-0.10 (-0.38- 0.19)
	Relief	-0.19 (-0.45- 0.11)
	Pliability	-0.13 (-0.41- 0.17)
	Surface	-0.21 (-0.47- 0.09)
	Overall impression	-0.09 (-0.37- 0.21)

Supplementary Table S4. Descriptive statistics for individual items of the Stony Brook Scar Evaluation Scale and SCAR-Q questionnaire. ICC = intra-class correlation coefficient, CI = confidence interval.

S4. Influence of passed time since surgery

	**Surgery <1 year ago (n=9)**	**Surgery ≥1 year ago (n=37)**	
	Mean ± SD	*Mean ± SD*	*p-value* *
Appearance	67.67 ± 17.89	75.92 ± 22.15	0.393
Symptom	60.89 ± 8.49	82.32 ± 15.10	<0.001
Psychosocial	79.11 ± 22.92	81.51 ± 24.36	0.760

Supplementary Table S5. Comparison of SCAR-Q domain scores between patients who underwent surgery <1 year ago versus those who underwent surgery ≥1 year ago. * Mann-Whitney-U test

REFERENCES

1. van de Kar AL, Corion LU, Smeulders MJ, Draaijers LJ, van der Horst CM, van Zuijlen PP. Reliable and feasible evaluation of linear scars by the Patient and Observer Scar Assessment Scale. Plast Reconstr Surg. 2005;116(2):514-22.
